# Reactive oxygen species formation and its effect on CD4^+^ T cell-mediated inflammation

**DOI:** 10.3389/fimmu.2023.1199233

**Published:** 2023-05-25

**Authors:** Panyin Shu, Hantian Liang, Jianan Zhang, Yubin Lin, Wenjing Chen, Dunfang Zhang

**Affiliations:** Department of Biotherapy, State Key Laboratory of Biotherapy and Cancer Center, Collaborative Innovation Center of Biotherapy, West China Hospital, Sichuan University, Chengdu, Sichuan, China

**Keywords:** reactive oxygen species, CD4^+^ T cells, inflammation, Treg cells, effector T cells (Teffs), Th17 cells

## Abstract

Reactive oxygen species (ROS) are produced both enzymatically and non-enzymatically *in vivo*. Physiological concentrations of ROS act as signaling molecules that participate in various physiological and pathophysiological activities and play an important role in basic metabolic functions. Diseases related to metabolic disorders may be affected by changes in redox balance. This review details the common generation pathways of intracellular ROS and discusses the damage to physiological functions when the ROS concentration is too high to reach an oxidative stress state. We also summarize the main features and energy metabolism of CD4^+^ T-cell activation and differentiation and the effects of ROS produced during the oxidative metabolism of CD4^+^ T cells. Because the current treatment for autoimmune diseases damages other immune responses and functional cells in the body, inhibiting the activation and differentiation of autoreactive T cells by targeting oxidative metabolism or ROS production without damaging systemic immune function is a promising treatment option. Therefore, exploring the relationship between T-cell energy metabolism and ROS and the T-cell differentiation process provides theoretical support for discovering effective treatments for T cell-mediated autoimmune diseases.

## Introduction

Reactive oxygen species (ROS) are oxidants produced during intracellular or extracellular aerobic energy metabolism, and enzymatic reactions have attracted extensive attention from researchers since their discovery (1, 2). The variety of ROS molecules is a collective term for several related molecules. ROS molecules that play physiological roles in the body include H_2_O_2_, ${\text{O}}_{2}^{-}$ , and OH-, and different single entities can be converted into one another through interconversion reactions (3–5). Initially, ROS were considered “toxic” molecules that destroy cellular components through oxidation, participate in the pathogenesis of various diseases, and lead to aging (6). Further research has shown that ROS have a dual function. The physiological concentration of ROS plays an important role as a regulatory medium in the signal transduction process, and metabolism-related diseases may be affected by the redox balance; however, when the concentration of ROS is uncontrolled, oxidative stress results in a disturbance in the normal redox state of the cell and/or oxidative damage (7–10). There are several processes through which ROS are generated *in vivo*, including those within the mitochondria (11), cytoplasm (12, 13), endoplasmic reticulum (ER) (14, 15), and peroxisomes (16, 17). Enzyme complexes can produce ROS, including NADPH oxidases and cytochrome P450-dependent oxygenases (18, 19).

T cell-mediated immune responses are essential for resisting multiple pathogenic microbial infections and antitumor immune responses (20). The core of the T cells activation process is metabolic reprogramming, in which oxidative phosphorylation is transformed into aerobic glycolysis (21–23). Because different CD4^+^ T-cell subtypes depend on different energy metabolism methods, T helper 1 (Th1), Th2, and Th17 cells rely on aerobic glycolysis to reduce lipid oxidation. Whereas regulatory T cells (Tregs) mainly depend on lipid oxidation as the main source of metabolism, ROS produced during oxidative metabolism affect the differentiation of CD4^+^ T cells through various mechanisms (24–30). In this study, we describe ROS, the ROS generation pathway *in vivo*, oxidative stress, and the harmful effects of oxidative stress on physiological functions. The main immune functions of different helper T cells, the energy source on which they depend for differentiation, and the effect of ROS produced during oxidative metabolism on the differentiation of CD4^+^ T cells are described. This study provides techniques for exploring new methods to treat autoimmune diseases.

## ROS and oxidative stress

ROS are byproducts of the redox reactions of oxygen molecules during biological oxidation. Because the ground-state oxygen molecule contains two unpaired electrons, it is readily reduced in redox reactions, and the product after reduction by a single electron can be used as a precursor to producing other ROS molecules. The term “reactive oxygen species” does not refer to a specific chemical molecule; it is a collective term that includes several related molecules with high chemical reactivity because of their unpaired electrons. This broad term ignores the fact that the biology of individual types of ROS is highly diverse, and their chemical reactivity and second-order rate vary significantly (3, 4, 31–33). The abundance of ROS in the body and the inherent duality of their functions have attracted the interest of numerous researchers over the past 50 years. Physiological ROS levels usually act as biological signals that regulate the physiological activities of organisms. However, supraphysiological concentrations of ROS lead to non-specific toxic effects on DNA, proteins, and lipids, causing damage to cellular and genetic structures (1, 2). Additionally, reactive nitrogen species (RNS), another common product of metabolism, have dual physiological functions similar to those of ROS (34). The commonly seen RNS is mainly the less reactive nitric oxide (NO), which can react with $O_{2}^{-}$ to form the highly oxidative peroxynitrite (ONOO^-^) (35, 36). As the major primary ROS species, H_2_O_2_, $O_{2}^{-}$ , and OH- play important roles in the redox regulation of biological activities (18, 37–51). Although each molecule in the ROS species can function biologically as a single entity, molecules can transform into others under certain reaction conditions. $O_{2}^{-}$ is decomposed to H_2_O_2_ by the action of superoxide dismutase (SOD), which can be further reduced to H_2_O or OH-. The process of OH- formation is accompanied by the oxidation of the [4Fe–4S] cluster. The generated iron is repeatedly reduced, allowing this process to continue (5, 52).

“Oxidative stress,” also known as oxidative adversity, describes a series of adaptive responses caused by the inability of the antioxidant system to remove excess oxidants promptly, causing alterations in the intracellular redox status, interfering with normal signaling, and mediating oxidative damage (2, 3). Oxidative stress is an embodiment of the dual functions of ROS (7, 8). When the steady-state concentrations of major ROS molecules, such as H_2_O_2_ and $O_{2}^{-}$ , are at a certain threshold, ROS can be used as versatile pleiotropic physiological signaling agents during the physiological activity of higher organisms (9, 38, 42). Oxidative modification of target proteins by ROS alters protein activity and localization, which regulates processes, such as signal transduction and metabolic metabolism, between cells and organs (10). Oxidation of nuclear factor-κB (NF-κB) by H_2_O_2_ can lead to its activation, but if T cells are exposed to H_2_O_2_ *in vitro* for a prolonged period, their DNA-binding capacity will be inhibited (53). Therefore, uncontrolled increases in the concentrations of these oxidants may lead to indiscriminate oxidative damage and altered response patterns in proteins, lipids, polysaccharides, and DNA, resulting in growth stagnation and death (8, 54–56). Research has shown that the pathogenesis of many diseases is linked to high concentrations of local ROS and oxidative damage, including cancer, diabetes, and neurodegenerative diseases (9, 10).

**Figure 1:**
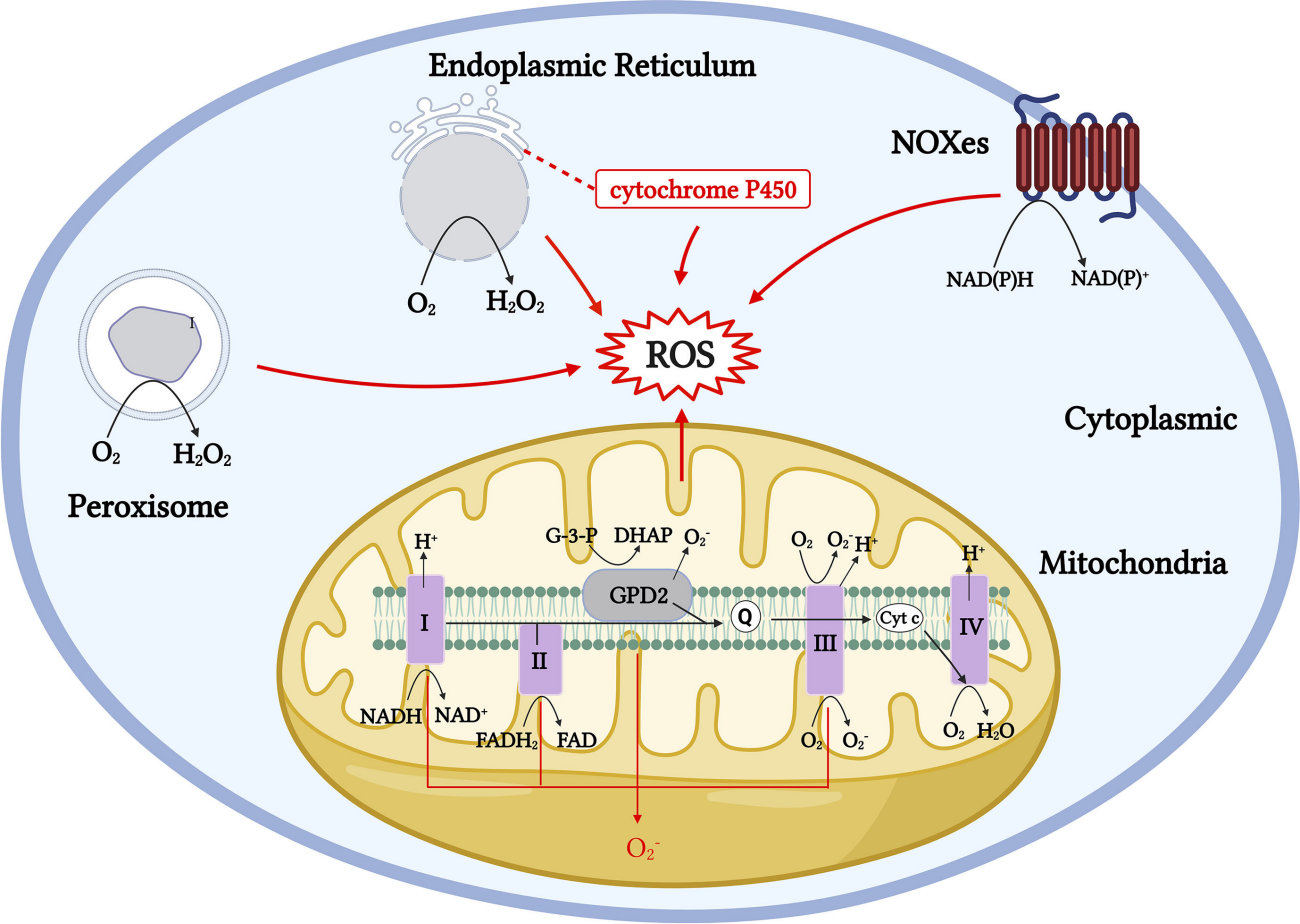
Generation pathway models of ROS production throughout the cell. Mitochondrial ROS are generated by numerous mechanisms, including Complexes I to III. Cytoplasmic ROS production mainly relies on the NADPH oxidase (NOX) family, and NOX proteins produce $O_{2}^{-}$ through NADPH electron exchange. The ER produces H_2_O_2_ by transferring acquired electrons through a flavin adenine dinucleotide cofactor to molecular oxygen. The membrane-associated monooxygenase system also produces ROS through cytochrome P450. Peroxisomes produce H_2_O_2_ from $O_{2}^{-}$ through oxidases, including ACOX and d-amino acid oxidase.

The duality of the roles of ROS is also evident in the physiological activity of T cells. Evidence shows that medium or low concentrations of ROS in T cells act as intracellular signaling molecules during homeostasis and antigen recognition. ROS levels and localization can alter the redox status of effector proteins and transcription factors (TFs), which could affect T-cell responses (45). However, high physiological concentrations of ROS can cause reversible and irreversible damage to cellular molecules and participate in the pathogenesis of numerous diseases. For example, physiological concentrations of ROS can participate in the activation and proliferation of T cells by activating TFs, such as NFAT, NF-κB, and AP-1 (57); however, if T cells are exposed to H_2_O_2_ for a long time *in vitro*, the DNA-binding ability of NFAT and NF-κB is selectively inhibited, resulting in the downregulation of IL-2 transcription (53). Long-term or chronic ROS upregulation can also lead to T-cell homeostasis disorders, mitochondrial membrane polarization, and T-cell failure and non-response (58, 59). Therefore, maintaining a stable physiological concentration of ROS in T cells or maintaining the redox balance is essential for the metabolism and function of T cells. Cells express a variety of antioxidant enzymes, such as SODs, catalase, peroxidase reductase, thioredoxin system (Trx), glutathione (GSH), and other small-molecule antioxidants to remove excess ROS, thereby maintaining the redox balance in cells (60–62). The same occurs in T cells. When the T-cell receptor (TCR) is stimulated, it is accompanied by the rapid production of a large amount of ROS. Simultaneously, antigen-presenting cells can secrete cysteine, which is absorbed by T cells to generate GSH, thereby avoiding the oxidative stress caused by the excessive production of ROS over a short period (63, 64). In the body, the main producers of ROS include localized and compartmentalized organelles and related enzymes, and the sources of ROS production *in vivo* can be divided into enzymatic and non-enzymatic pathways (9, 10, 38) (**Figure 1**). Mitochondria are the main source of aerobic energy in eukaryotes. The electron transport chain (ETC) is a continuous reaction system consisting of four membrane protein complexes and lipid-soluble electron carriers in a certain order, used to convert the reduction potential into a proton gradient across the membrane, accompanied by electron transfer to bind to oxygen molecules and produce water and generate adenosine triphosphate (ATP) for energy. ROS are produced by the electrons that “leak” from respiratory chain complexes (11). In addition to the mitochondria, ROS are byproducts of other cell compartments, including the cytoplasm, cell membrane, ER, and peroxisomes. Enzymatic sources include NADPH oxidases (nicotinamide adenine dinucleotide phosphate, NOX), located on the cell membrane of polymorphonuclear cells, macrophages, endothelial cells (ECs) (47, 48, 65, 66); cytochrome P450-dependent oxygenases (18, 19); monoaminoxidase (MAO) (67, 68); a-glycerophosphate dehydrogenase (69, 70); electron transfer flavoprotein (ETF), and ETF quinone oxidoreductase (ETF dehydrogenase) (71, 72) (**Figure 1**). Several major ROS production mechanisms are described in the following section.

## Generation pathway of ROS in cells

Mitochondria-derived ROS are mainly generated by membrane protein Complexes I and III and lipid-soluble electron carriers through various complex mechanisms in the mitochondria. The electrons used to generate ROS enter the respiratory chain, starting with Complex I (NADH-ubiquinone oxidoreductase) to produce a large amount of $O_{2}^{-}$ . Complex I mainly depends on two mechanisms: when the matrix NADH/NAD^+^ ratio is high, the flavin mononucleotide (FMN) site on Complex I is reduced, and when the ubiquinone pool is over-reduced, the reduction potential of the ubiquinone/ubiquinol redox pair favors the reduction of Complex I at the Q binding site (site I_Q_) (73, 74). Complex II and mitochondrial glycerol 3-phosphate dehydrogenase (mGPDH; GPD2) have been shown to drive RET and produce mitochondrial ROS from Complex I (75–78). O_2_ interacts with reduced FMN to generate $O_{2}^{-}$ and $O_{2}^{-}$ produced in Complex I, which is released into the mitochondrial matrix and converted to H_2_O_2_ by manganese superoxide dismutase (MnSOD). As a major component in the production of mitochondrial-derived ROS, the impaired function of Complex I leads to excessive superoxide production and is involved in the pathogenesis of Parkinson’s disease (PD) and various neurodegenerative diseases. Parkinson-related mutations lead to increased production of mitochondrial superoxide and other ROS, in addition to localized high ROS concentrations in this region because of a lack of GSH in the substantia nigra, making it vulnerable to oxidative damage (79–82). The reducing equivalents formed in Complexes I, II, and GPD2 are passed to the Q-cycle of Complex III (ubiquinone-cytochrome c oxidoreductase) for further processing, where GPD2 catalyzes the unidirectional conversion of glycerol-3-phosphate (G-3-P) to dihydroxyacetone phosphate (DHAP) (78) (**Figure 1**). Complex III emits $O_{2}^{-}$ into the matrix and intermembrane space. The formation of $O_{2}^{-}$ in Complex III can enter the cell membrane from the intermembrane space through voltage-dependent anion channels without prior conversion to H_2_O_2_, unlike Complex I, which requires converting $O_{2}^{-}$ to H_2_O_2_ before release into the mitochondrial matrix. Studies have shown that the production of $O_{2}^{-}$ in Complex III is much lower than that in Complex I and is therefore negligible under physiological conditions (11, 83, 84). Complex II, often referred to as succinate-coenzyme Q reductase, uses succinate to reduce coenzyme Q to QH2 using covalently bound FAD as a coenzyme to produce reduced flavin adenine nucleotides (FADH2) (**Figure 1**). In addition to mitochondrial ROS from Complexes I, II, and III, enzymes, such as the α-ketoglutarate dehydrogenase (KGDHC) and pyruvate dehydrogenase (PDC) complexes, involved in mitochondrial metabolism also produce ROS through forward electron transfer, and both act as important sources of ROS in the mitochondria (85–87) (**Figure 1**).

**Figure 2:**
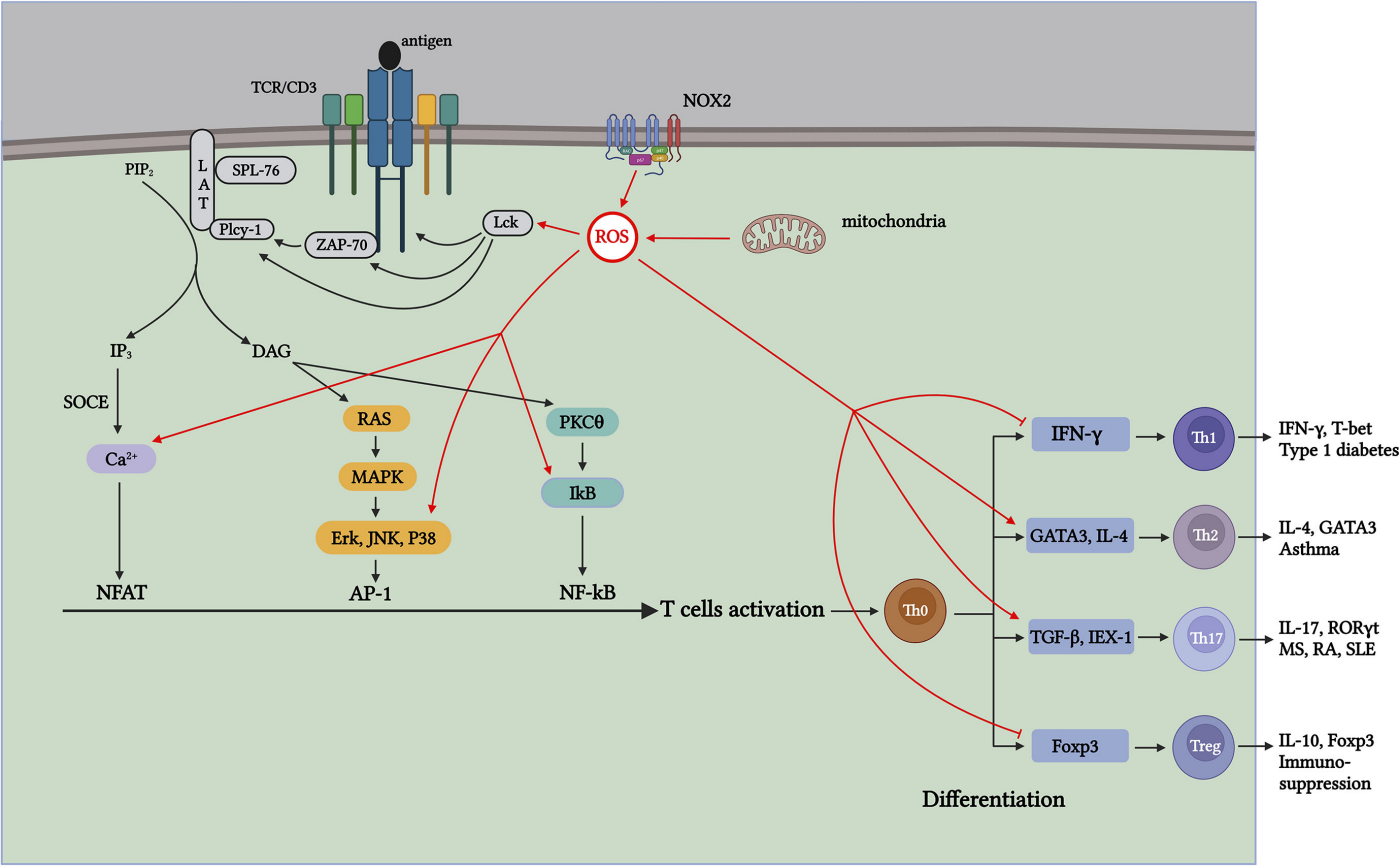
ROS generated during T-cell activation affects TCR signaling and CD4^+^ T-cell differentiation through distinct mechanisms. Antigen-stimulated TCR induces mitochondria and NOX2 to produce ROS, which promotes T-cell activation and proliferation by further activating signaling molecules in the TCR signaling pathway. Th1 cells contribute to type 1 diabetes (T1D); Th17 cells contribute to pathogenesis, including multiple sclerosis (MS), RA, and systemic lupus erythematosus (SLE); Th2 cells participate in the development of asthma; and Treg cells exert suppressive immune regulation. ROS produced during aerobic glycolysis affects the activation and differentiation of CD4^+^ T cells through various mechanisms, thus affecting the process of autoimmune diseases mediated by different T-cell subsets.

Cytoplasmic ROS regulates the pentose phosphate pathway (PPP), glycolytic pathways, and other physiological activities. The NADPH oxidase (NOXes) family is a major source of cytoplasmic ROS, and NOX-dependent ROS production is involved in many physiological and metabolic activities and disease pathogeneses (12, 13). ROS produced by NOX are essential for the oxidative burst, in which several innate immune cells kill engulfed pathogens (44). NOX2 and DUOX1 are likely the major NOX isoforms in T cells, and once TCR is stimulated, NOX 2 transfers electrons to oxygen to produce $O_{2}^{-}$ (88) (**Figure 2**). In the ER, protein folding is highly sensitive to changes in redox homeostasis and is one of the main sources of H_2_O_2_ production. H_2_O_2_ is the main ROS molecule produced by the ER. In the ER, ER oxidoreductin 1 (ERO1) accepts electrons from peptide substrates via protein disulfide isomerases (PDI) and transfers them for molecular oxygen generation to produce H_2_O_2_ (14, 15, 89, 90). A large proportion of aerobic metabolism in the body is conducted with the involvement of the peroxisome, which transfers hydrogen from substrates to O_2_ to produce H_2_O_2_ through a variety of oxidases. The types of oxidases that function in different tissues vary markedly (16, 17, 91) (**Figure 1**).

## Role of ROS in activation of CD4^+^ T cells

T cells are central to immune system function. They mediate adaptive immune responses, complement the humoral immune response, and develop immune tolerance to autoantigens. T cells are also essential for specific defense against pathogenic microorganisms. Quiescent lymphocytes and monocytes require minimal energy compared to their activated counterparts, and these demands are met primarily by oxidative phosphorylation. When resting naïve T cells are stimulated by antigens to meet the energy required for T-cell activation, they undergo metabolic reprogramming to enhance activity, biosynthesize intermediates, and construct signaling molecules to spread anabolism, thereby initiating the rapid proliferation and differentiation of CD4^+^ T cells (92). When an antigen stimulates TCR, it binds to the co-stimulatory molecule CD28 and secretes cytokines, such as IL-2, which induce the activation of TF Akt. Activated Akt initiates the mammalian rapamycin (mTOR) pathway, reprogramming energy metabolism from oxidative phosphorylation to glycolysis. These changes increase nutrient uptake and glucose metabolism rates, promoting the activation and proliferation of naïve T cells (21, 22, 93). Additionally, Myc participates in upregulating aerobic glycolytic metabolism in T cells to meet energy demands for activation and proliferation (93). Therefore, the energy source for CD4^+^ T-cell differentiation mainly relies on aerobic glycolysis; however, energy sources for differentiating different CD4^+^ T-cell subsets are also distinct. T helper cells depend on aerobic glycolysis and reduce lipid oxidation, whereas Tregs mainly depend on fatty acid oxidation (FAO) as the main source of energy for metabolism (23–30, 93–99) (**Figure 2**).

ROS are the byproducts of oxidative metabolism. Owing to the increased demand for energy metabolism during T-cell activation, the local concentration of ROS increases rapidly within a short period (100). ROS produced during T-cell activation have two main physiological sources: mitochondria and the NADPH oxidase complex (88, 101). As a major source of ROS, the mitochondrial ETC transfers electrons from NADH and FADH2 to Complex IV to generate water. During the electron transfer process, a small portion of the “leaked” electrons can react non-enzymatically with O_2_ to generate $O_{2}^{-}$ (102, 103) (**Figure 1**). Studies have shown that when the functional activity of the respiratory chain Complex III is specifically inhibited in T cells, the energy generated by mitochondrial respiration is insufficient for the activation of T cells and the secretion of sufficient cytokines IL-2 and IL-4 (100, 104–108). Continuous treatment of pre-activated primary human T cells with ciprofloxacin inhibited TCR-induced ROS production and IL-2 and IL-4 expression, and this inhibitory effect was significantly correlated with the dose of ciprofloxacin. Studies have shown that ROS production in Complex I in resting and pre-activated human T cells is essential for activation-induced IL-2 and IL-4 expression and secretion when TCR is stimulated (109). Studies have shown that the production of mROS is significantly limited when T cells lacking Complexes I and III are activated, resulting in the decreased expression and proliferation of IL-2 and IL-4, which affects the activation process (109). Therefore, the results show that mitochondrial metabolism, especially the production of ROS in mitochondrial Complexes I and III, is important for T-cell activation. In addition to the mitochondria, the NOX protein family is a major ROS producer during CD4^+^ T-cell activation (88). The NOX enzyme family consists of seven members (NOX 1–5 and two dioxygenases [DUOX], namely 1 and 2). In T cells, TCR-induced ROS production increases mainly depend on two major NOX subtypes: the phagocyte subtype NOX2 and the non-phagocyte subtype DUOX1 (88, 110). The mROS produced by TCR upon antigen stimulation can activate NOX2, which maintains intracellular ROS levels, thereby promoting T-cell activation and proliferation (102). Studies have shown that the lack of NOX2 leads to a significant decrease in $O_{2}^{-}$ and H_2_O_2_ in T cells; however, the reduction in NOX2-derived ROS has little effect on T-cell activation and proliferation. Therefore, whether the ROS generated from NOX2 play a crucial role in T-cell activation requires further investigation (111) (**Figure 2**). DUOX1 is also a component of the redox signal after TCR stimulation, and inhibition of DUOX1 expression significantly reduces anti-CD3-mediated H_2_-DCFDA oxidation (112, 113).

When stimulated by antigens, TCR signaling is triggered in the plasma membrane, leading to IL-2 production, which further drives the activation and proliferation of T cells. ROS produced during this process, in turn, act as key signaling molecules that regulate the activation of T cells (107, 108, 114). The immunoreceptor tyrosine-based activation motifs (ITAMs) in the cytoplasmic region of the TCR contain two core tyrosines. Tyrosine lymphocyte-specific protein tyrosine kinase (Lck) phosphorylation in ITAM is activated after antigen stimulation (115). Studies have shown that T-cell development is notably blocked in Lck-specific knockout mice (116). Simultaneously, phosphorylated ITAM is further activated by recruiting ZAP-70, and the lack of ZAP-70 can significantly affect signal transduction downstream of the TCR (117, 118). The binding of ZAP-70 to ITAM also activates ZAP-70. Activated ZAP-70 forms a complex signal skeleton by phosphorylating LAT and SLP-76 for signal diversification (119). The formation of the LAT signaling complex activates PLCγ-1 to produce the second messengers diacylglycerol (DAG) and inositol 3,4,5-triphosphate (IP3). DAG activates NF-κB and initiates the Ras-ERK signaling cascade pathway, activating AP-1 (120, 121). Activation of the receptor of the second messenger IP3 leads to the storage of Ca^2+^ entry (SOCE) in the ER membrane, and the influx of Ca^2+^ signal transduction activates the TF NFAT (122, 123). At the transcriptional level, the activities of TFs NFAT, AP-1, and NF-κB induce the expression of IL-2 mRNA, which promote T-cell activation and proliferation (124) (**Figure 2**). Various signaling molecules in the TCR signal transduction pathway contain cysteine residues sensitive to oxidation. Therefore, ROS generated during activation affects TCR signal transduction after antigen stimulation. ROS inducers can promote the formation of lipid rafts on the plasma membrane, which contain important molecules involved in TCR signaling, such as LAT, PLCγ1, and PKCθ (125) (**Figure 2**). The activation of AP-1 requires the regulation of MAPK members, whereas the phosphorylation of Erk, JNK, and P38, which are important components of the MAPK pathway, depends on H_2_O_2_ (126, 127) (**Figure 2**). Because tyrosine kinases can regulate IκB (an NF-κB inhibitor) (128), H_2_O_2_ can indirectly regulate the TF NF-κB activation via tyrosine kinase Lck and ZAP-70, thereby activating gene transcription (129, 130) (**Figure 2**). In addition, research has shown that ROS produced in the mitochondria during T-cell activation affects Ca^2+^ homeostasis in a concentration-dependent manner, activating the TF NFAT (107, 131). Therefore, ROS production upon antigen stimulation of TCR promotes TCR signaling and transcriptional activation of IL-2 (**Figure 2**).

Because CD4^+^ T cells have different subtypes, the energy metabolism for the proliferation and differentiation of different subtypes also varies. Next, we describe the effects of mitochondrial ROS production on differentiating CD4^+^ T cells.

## Role of ROS in CD4^+^ T-cell differentiation

Within the CD4^+^ T-cell subpopulation, Th1 cells were among the first cells identified to be involved in the immune response against foreign antigens, such as microbes. They play an important role in mediating cellular immunity through the secretion of IL-2, TNF-α, and other cytokines and in clearing intracellular pathogens through the secretion of IFN-γ and lymphotoxins. Additionally, Thl cells are involved in the pathogenesis of many autoimmune diseases *in vivo*, with T1D being the most notable (132). Th1 cells induction begins with antigen-presenting cells (APCs) secreting IL-12, and the IL-12 induces natural killer cells (NK) to produce cytokine IFN-γ. The differentiation process of Th1 cells is tightly regulated by a feedback loop, with Th1 differentiation mainly regulated by TF T-bet, which can promote IFN-γ secretion by upregulating IL-12R*β*2 receptor expression and T-bet expression regulated by signal transducer and activator of transcription 1 (STAT1), which is in turn activated by IFN-γ secreted by TH1 cells (133). When the feedback loop in activated T cells is disrupted, reduced IL-12 secretion decreases that of IFN-γ and T-bet, thereby affecting the Th1 cell-mediated immune response (134–140).

Insulin is a key hormone secreted by B cells that can promote glucose uptake and glycolysis in the liver and muscle cells by converting glucose into glycogen, thereby reducing blood glucose concentration in response to high glucose. A decrease in insulin levels causes excessive blood glucose concentrations, leading to the occurrence and development of diabetes (141). It was demonstrated that Th1 cells with the specific diabetic TCR could induce T1D in NOD mice, and the Th1 cells marker IFN-γ is directly involved in the process of T1D diseases (142). Research shows that IFN-γ can participate in the progress of T1D diseases through several pathways, which include mediating beta cell death by stimulating the cytotoxic CD8 T cells response (142). Therefore, constraining IFN-γ responses (e.g., enhancing Treg function and/or inhibiting Th1 cells differentiation/function) may prevent the onset of diabetes (143–147).

Differentiation of Th1 cells primarily relies on aerobic glycolysis to promote the secretion of T-bet and IFN-γ. Research has shown that in Glut1-deficient Teff cells, owing to their decreased glucose transport capacity, the resulting Th1 cells decreased and showed less pronounced colitis symptoms in mouse models. As a necessary regulator that promotes the differentiation of Th1 cells, studies have revealed that lactate dehydrogenase A (LDHA) is a key factor supporting aerobic glycolysis and promoting IFN-γ expression, driving naïve T cells to differentiate into Th1 cells. In this study, the elimination of LDHA in T cells resulted in the inhibition of IFN-γ overexpression in mice, restoration of normal Treg cell function, and reduction in immunopathological damage (148–150). Another relevant study showed that H_2_O_2_ enhances IL-4 production, downregulates IFN-γ production, and promotes the naïve T cells into the Th2 lineage without altering cell proliferation (151, 152). Therefore, reducing autoreactive Th1-type CD4^+^ T-cell glycolysis and/or inhibiting antioxidant activity could be potential strategies to prevent the development of T1D (**Figure 2**).

In contrast to the two classical lineages, Th1 and Th2 cells, IL-17-producing T helper 17 (Th17) cells have been classified as an important emerging inflammatory effector CD4^+^ T-cell subset. Th17 cells cause chronic tissue inflammation and organ failure (153–156). The differentiation of Th17 cells is mainly accomplished by the involvement of IL-6, IL-23, IL-21, and transforming growth factor-β (TGF-β), with TGF-β being the critical cytokine for Th17 differentiation (157–160). Th17 cells stimulate tissue cells to secrete antimicrobial peptides, enhance the immune barrier function of epithelial tissue, and stimulate the local production of cytokines, such as chemokines, which induce an inflammatory response dominated by neutrophils and monocytes (161, 162). In addition, many studies have shown that pro-inflammatory (pathogenic) Th17 cells are involved in the pathogenesis of a variety of inflammatory and autoimmune diseases, such as multiple sclerosis (MS), RA, psoriasis, and inflammatory bowel disease (IBD). Stimulated neoplastic T cells destroy myelin sheaths and axons by generating a pro-inflammatory response, thereby inducing experimental autoimmune encephalomyelitis (EAE), a common experimental model used to study MS (163–166).

Similar to that in Th1 cells, aerobic glycolysis is the energy source for Th17 cell differentiation. The increase in aerobic glycolysis during T-cell activation results in a significant increase in the transport of glucose and amino acids for biosynthesis and energy supply, thus highlighting the importance of Glut 1. Studies have shown that Glut 1 deficiency reduces the efficiency of glycolysis, which in turn affects T-cell activation and proliferation, and a significant number of cells undergo apoptosis (150). IEX-1 affects the proliferation, differentiation, and survival of these cells by accelerating ATP hydrolysis, which in turn impedes ROS production. It has been shown that IEX-1 deficiency promotes the differentiation of Th17 cells. This process is mediated by increased mitochondrial ROS production (167). In addition, research has shown that the upregulation of ROS produced by oxidative metabolism during T-cell activation promotes the differentiation of Th17 cells by activating TF TGF-β. In this study, high glucose-induced upregulation of mitochondrial ROS drives Th17 cell differentiation by activating TGF-β and exacerbating autoimmunity in a mouse model of colitis and EAE (168). Therefore, targeting the immune metabolism to treat Th17 cell-mediated autoimmune diseases has broad application prospects (169) (**Figure 2**).

Th2 cells, another traditional genealogy in the subset of CD4^+^ T cells, are naïve T cells that differentiate into Th2 cells by producing IL-4 by inducing the expression of the key TF Gata3. Th2 cells can express lineage-defining TFs, GATA3 and STAT6. GATA3 contributes to the Th2 phenotype by inducing IL-4 to form a positive feedback loop (170–173). However, no known Th2-mediated autoimmune diseases involving Th1 or Th17 cells exist. Th2 cells assist in the proliferation and differentiation of B cells into plasma cells by secreting cytokines, such as IL-4, IL-5, and IL-13, inducing the transformation of macrophages into the M2 phenotype and the recruitment of eosinophils, thereby protecting against worms, poisons, and certain bacteria, and stimulating tissue healing (174–179). In addition, Th2 cells are involved in the immune response and pathogenesis of allergic diseases, including asthma and atopic dermatitis. Disease progression in animal models of RA can be influenced by modulation of the balance between Th1 and Th2 cells. Therefore, the Role of Th2 cells in autoimmune diseases has received increasing attention (180–183).

Differentiation into Th2 cells is dependent on mTOR activity, which is reduced when mTOR activity is inhibited by rapamycin (184, 185). Moreover, the anti-CD3 antibody can reduce ROS production by mitochondrial Complex I and inhibit the expression of IL-2 and IL-4, thereby inhibiting Th2 cell differentiation (109). Research has confirmed that by downregulating the synthesis of superoxide anions and nitric oxide, IL-4 and IL-13 can be reduced, and IL-1β production increased, transforming Th2 into a Th1 response (**Figure 2** ). Thus, avoiding airway hyperresponsiveness (AHR) in an asthma model effectively improves the symptoms of patients with asthma (186, 187).

Th1, Th2, and Th17 cells directly or indirectly mediate autoimmune diseases and elicit immune responses against foreign fungi, parasites, and other infections. Tregs are key regulators of inflammation and autoimmunity. They can exert negative immune regulation through various mechanisms important for maintaining self-tolerance and immune homeostasis in multiple tissues, thereby avoiding excessive damage to the body from immune responses. Unlike other CD4^+^ T-cell subsets, the energy source for Treg cells differentiation rely mainly on FAO rather than aerobic glycolysis (28–30, 188). The differentiation conditions for Treg cells were similar to those for Th17 cells. TGF-β is required for the differentiation of both Th17 and Treg cells. Treg cells can be induced when only TGF-β is present in the culture conditions, while TGF-β and IL-6 preferentially induce Th17 cells. Studies have shown that IL-6 can increase glucose metabolism by promoting glucose and glycogen binding and glucose oxidation in skeletal muscles, whereas the differentiation of Tregs mainly relies on FAO (28–30, 189–193). Foxp3 is a key lineage-defining TF for Treg cells that inhibits the differentiation of naïve T cells toward Th17 cells by limiting RORγt activity and, together with other regulators, maintains the development and function of Treg cells (194–198). Although Tregs are not directly involved in the immune response to foreign antigens, they are necessary for maintaining immune tolerance to autoantigens and immune homeostasis *in vivo*. The exhaustion of Treg cells in the body can lead to severe autoimmune diseases, and the lack of CTLA-4 is the key molecule causing this problem (199–202). Tregs exert suppressive immune regulation through multiple mechanisms, including the secretion of IL-35, IL-10, and other soluble suppressive immune molecules, to suppress effector signals directly. Tregs can produce high-affinity IL-2 receptors that bind IL-2 competitively with T cells, inhibiting the proliferation and apoptosis of activated T cells. Tregs can also induce apoptosis in a perforin-dependent manner via granzymes A and B (201, 203–207).

Unlike Teffs, which depend on aerobic glycolysis for differentiation, Tregs rely on FAO as their energy source (28–30). Foxp3 expressed by Treg cells can bind to the Myc promoter and suppress Myc gene expression to inhibit glycolysis, thereby stabilizing function and activity of Treg cells (93). Therefore, inhibiting the activity of aerobic glycolysis, such as inhibiting the activity of the glucose transporter Glu1, will seriously impact the differentiation of Teffs but will not affect the activity and function of Treg cells. Tregs can also use this feature to treat autoimmune diseases (208, 209). Studies have shown that inhibiting ROS production during oxidative metabolism reduces the suppressive effect of ROS on Treg cells, thereby regulating Th17/Treg cells and effectively improving psoriasis symptoms in mice (210) (**Figure 2**).

## Conclusion

ROS are byproducts of aerobic metabolism. Studies related to immune metabolism have shown that T cell-derived ROS and immune metabolic reprogramming further affect the outcome of the activation and differentiation of naïve T cells. Studies have shown that the pathogenesis of numerous autoimmune diseases is strongly correlated with CD4^+^ T cells and mitochondrial dysfunction, which leads to oxidative stress and may affect disease progression by altering CD4^+^ T-cell status, thereby interfering with normal therapeutic strategies and causing unexpected suffering in patients. Therefore, studying the specific effects of ROS in autoimmune diseases has significant implications for exploring more effective treatments. Current treatment options include global immunosuppression, immune-depleting antibodies, and anti-cytokine therapies. Although these treatments primarily target immune cells involved in pathogenesis, they inevitably damage normal functional cells in the body, leading to increased susceptibility to other diseases and complications. Because CD4^+^ T-cell activation and T helper cells differentiation are highly dependent on aerobic glycolysis, inhibition of autoreactive T-cell activation and T helper cells differentiation by targeting glycolysis or ROS generation without damaging systemic immune function is a promising direction for solving the problems of current treatment options. Many studies have been conducted using optimized *in vitro* cell culture media; however, it remains unknown how the metabolic microenvironment in healthy or disease-affected organs affects ROS production *in vivo* and, consequently, T-cell activation and differentiation. The Role of ROS in the effector function of T cells *in vivo* needs to be fully explored to exploit this property for better disease treatment. Antioxidants, such as GSH and SOD, can specifically remove oxidants, such as ROS, and maintain the redox balance in the body, thereby effectively preventing the damage caused by oxidative stress. Therefore, using antioxidants as a breakthrough in treating autoimmune diseases by targeting ROS has received extensive attention. However, because GSH cannot cross the blood-brain barrier and has a short half-life, oral administration of GSH does not significantly improve disease progression. Therefore, if the key issue of efficiently using antioxidants can be addressed, it will provide a new immunotherapeutic approach to suppress T cell-mediated autoimmune diseases. Improving self-tolerance by promoting Treg differentiation is an alternative therapy for autoimmune diseases.

## Author contributions

PS drafted the manuscript. HL edited the manuscript. JZ, YL, and WC revised the manuscript. DZ supervised the study and edited the manuscript. All the authors contributed to the study and approved the manuscript for publication.
